# Supplementary material for: Evaluation of statistical approaches for association testing in noisy drug screening data
Source: BMC Bioinformatics. 2022 May 18;23:188. doi: 10.1186/s12859-022-04693-z (PMC9118710; doi:10.1186/s12859-022-04693-z)
Supplement: Supplementary file 1 — Additional file 1. Supplementary Figures S1–S5. [file 12859_2022_4693_MOESM1_ESM.pdf]

Supplemental Figures

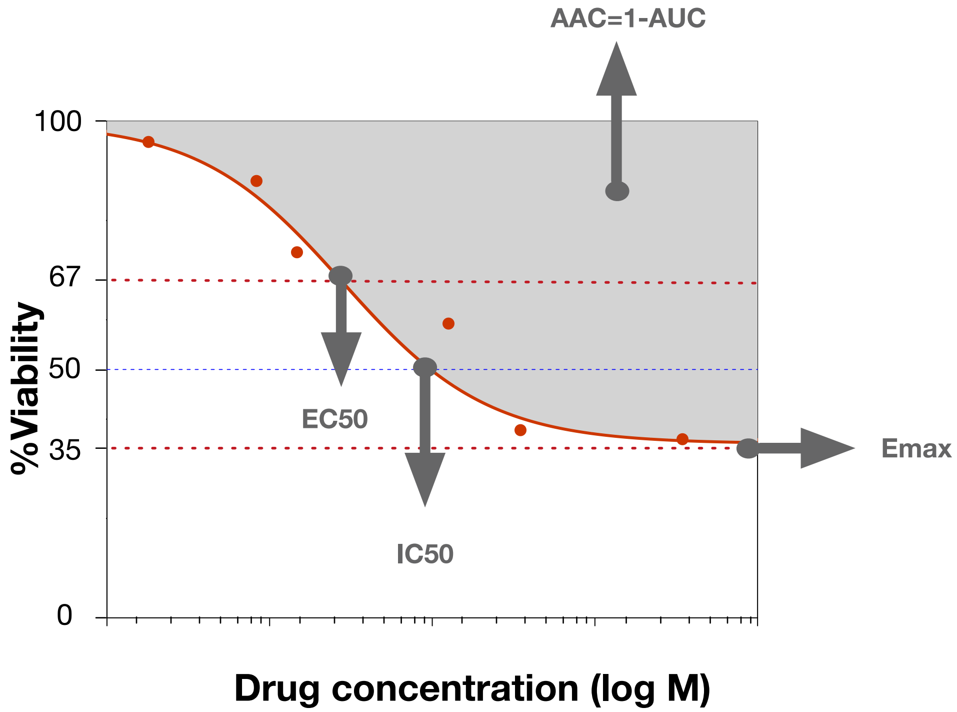

Figure S1: A prototypical drug dose - viability curve, including a representation of various metrics which can be calculated to summarize the curve behaviour. Reproduced from Safikhani et al. [1].

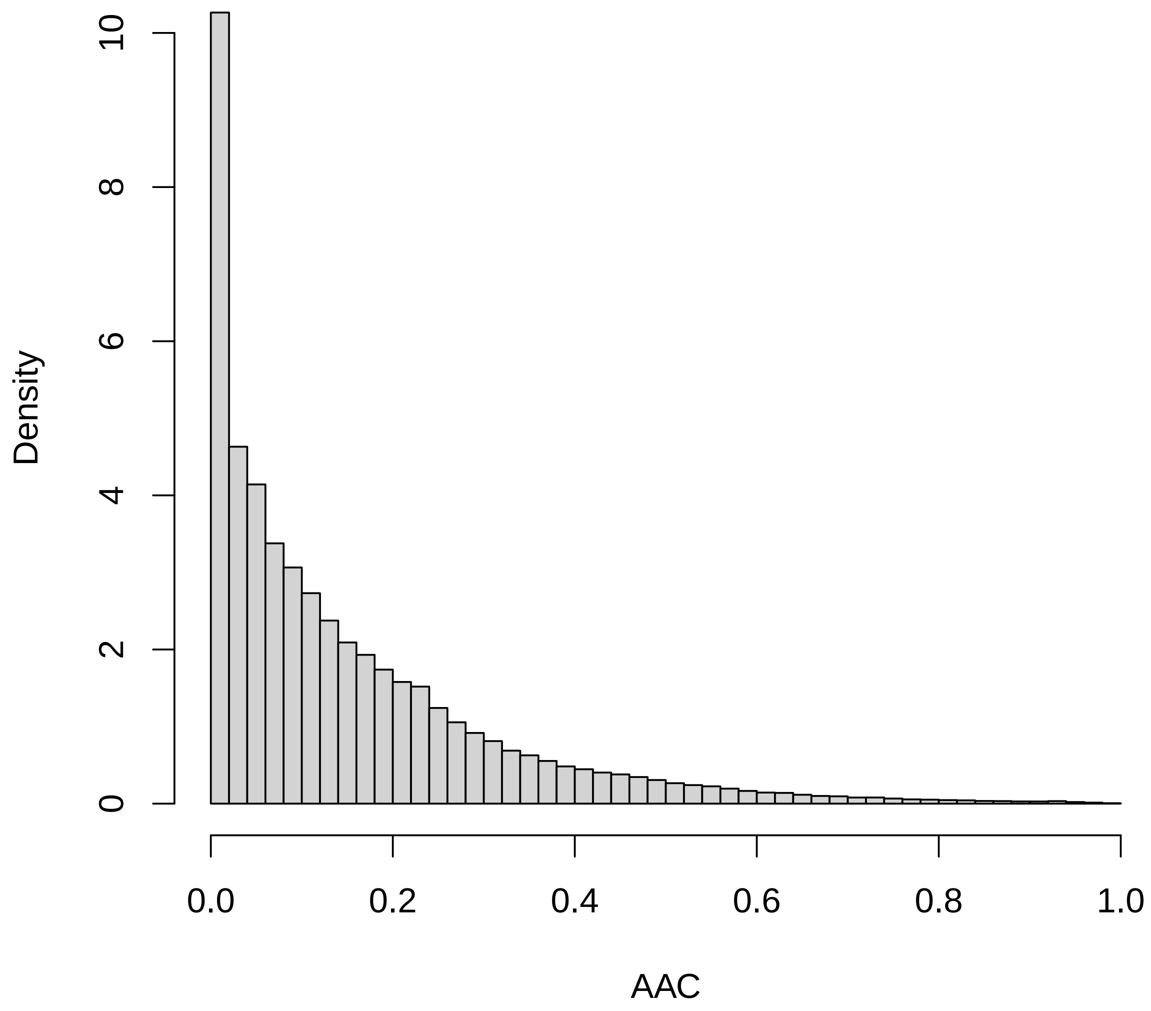

Figure S2: The distribution of all AAC values investigated in this study, combining across cell lines, drugs and studies. We can see the bounded and skewed behaviour which is simulated by a Beta distribution in our work.

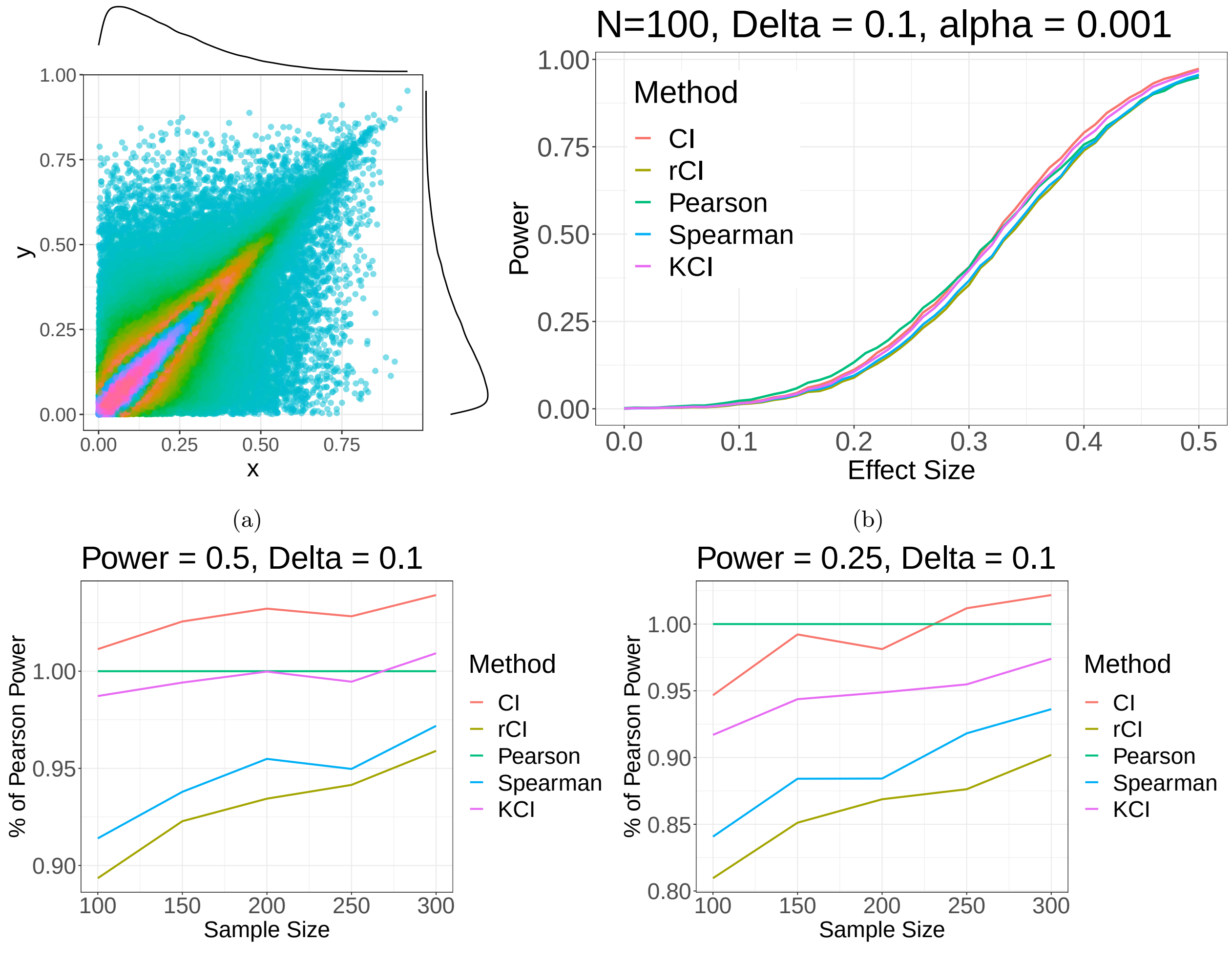

(a) (b) (c) (d)

Figure S3: Results of power analysis simulating Beta(1.2,4.5) distributed data. Panel (a) shows 10,000 samples from a member of the family of distributions used to sample data, in this case with correlation of 0.7 between the two variables. Panel (b) shows the observed power in simulation as a function of effect size, for simulated data of length 100. The Pearson correlation is most powerful at lower effect sizes, while the CI for effects were the power observed is 0.5 or larger. As the sample size is increased while decreasing effect size to target a constant Power for Pearson, the CI gains in power relative to the Pearson correlation, both for regimes of moderate (0.5, Panel (c)) and low power (0.25, Panel (d)).

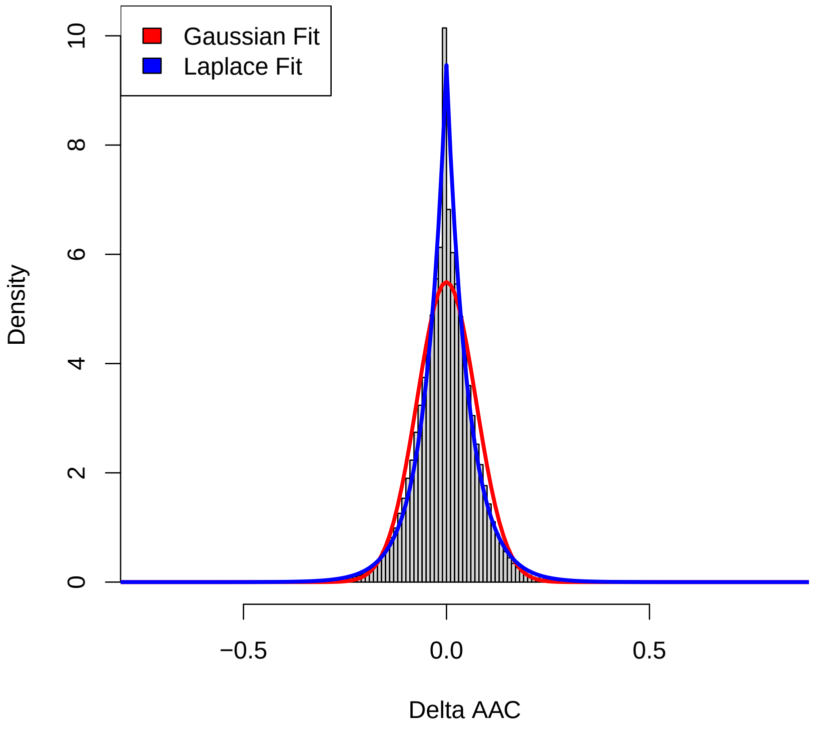

(a)

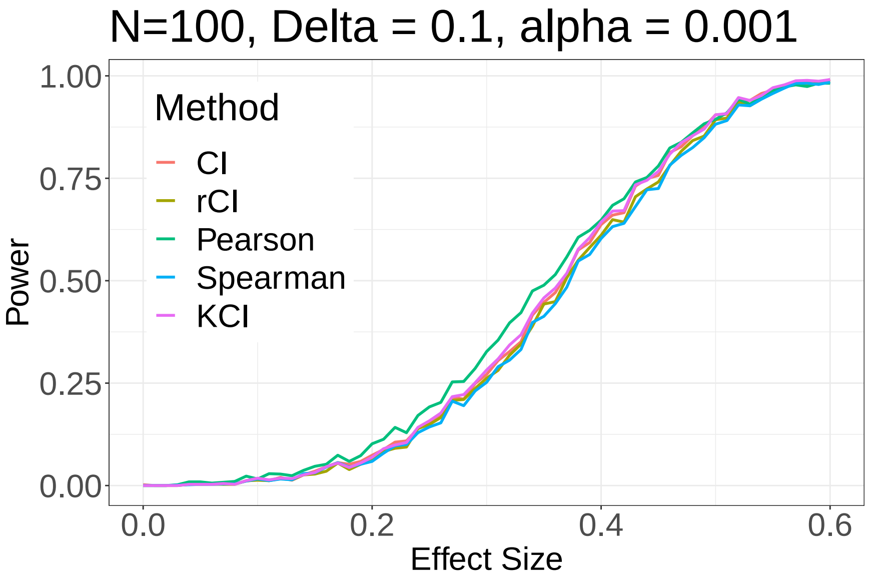

(b)

Figure S4: The distribution of differences in AAC measurements between replicates across all the datasets (Delta AAC) (a), with the MLE Gaussian and Laplace distribution plotted over the histogram of observed values. Power simulations for Beta distributed data were repeated with noise sampled from the MLE laplacian to one of the vectors added to the data, for expected correlations prior to adding noise of 0-0.6. While all correlation coefficients displayed lower power than in the noise-free case, surprisingly the Pearson correlation was least affected.

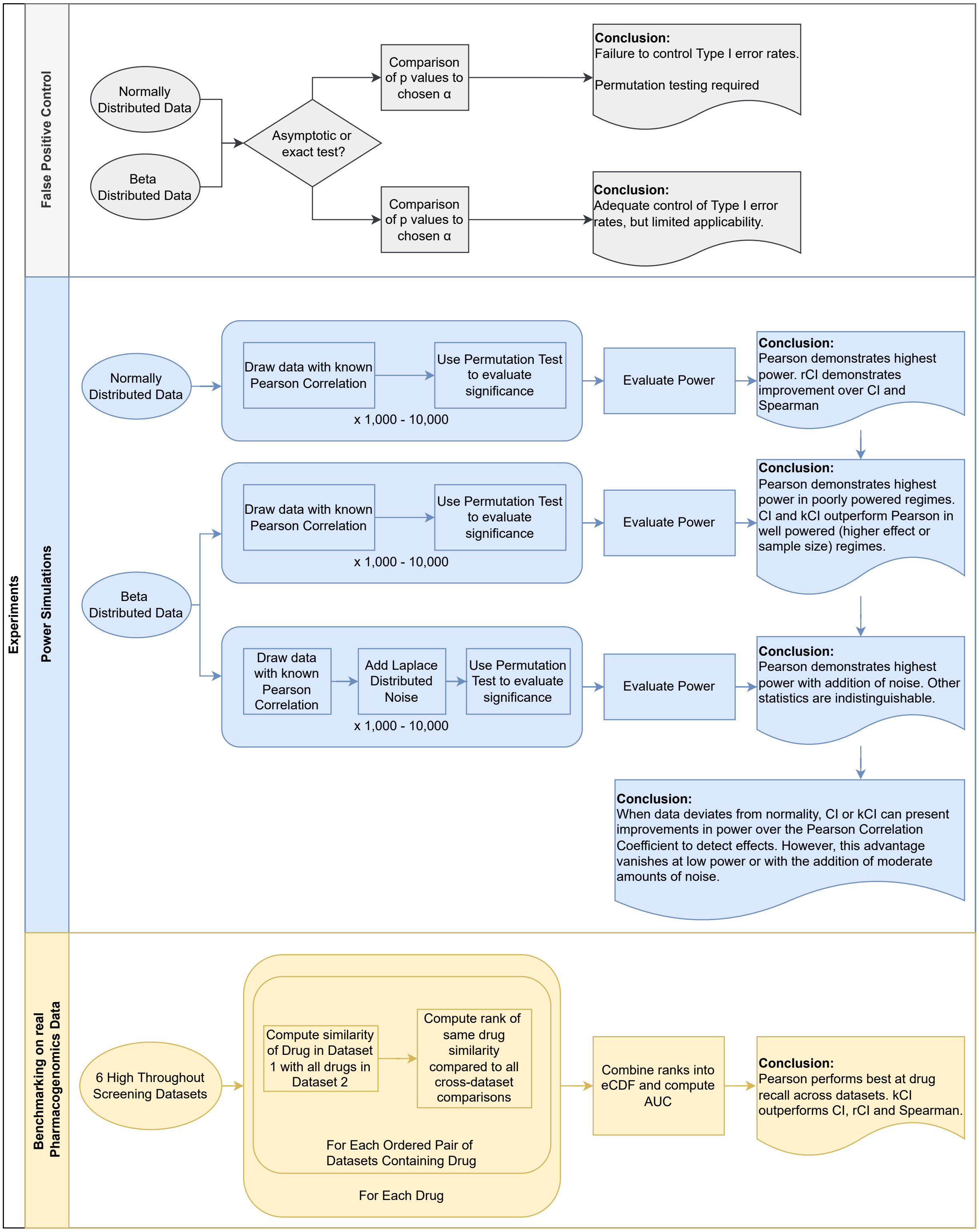

Figure S5: A diagram summarizing the experiments performed in this work and the conclusions derived from them. Experiments are organized into three main categories: those investigating Type I error control, those investigating Power in Simulation, and those Benchmarking performance on real pharmacogenomic data.
